# Medicine is Patriarchal, But Alternative Medicine is Not the Answer

**DOI:** 10.1007/s11673-018-9890-5

**Published:** 2018-12-20

**Authors:** Arianne Shahvisi

**Affiliations:** Lecturer in Ethics and Medical Humanities, Brighton & Sussex Medical School, University of Sussex, Falmer, BN1 9PX UK

**Keywords:** Alternative medicine, Women’s health, Autonomy, Informed consent, Feminism

## Abstract

Women are over-represented within alternative medicine, both as consumers and as service providers. In this paper, I show that the appeal of alternative medicine to women relates to the neglect of women’s health needs within scientific medicine. This is concerning because alternative medicine is severely limited in its therapeutic effects; therefore, those who choose alternative therapies are liable to experience inadequate healthcare. I argue that while many patients seek greater autonomy in alternative medicine, the absence of an evidence base and plausible mechanisms of action leaves patients unable to realize meaningful autonomy. This seems morally troubling, especially given that the neglect of women’s needs within scientific medicine seems to contribute to preferences for alternative medicine. I conclude that the liberatory credentials of alternative medicine should be questioned and make recommendations to render scientific medicine better able to meet the needs of typical alternative medicine consumers.

## Introduction

Women dominate alternative medicine (AM), both as consumers (Sharma 1992, 19–20; Ernst and White 2000; Cherkin et al. 2002; Tindle et al. 2005; Verhoef et al. 2005; Eardley et al. 2012) and as service providers (Cant and Sharma 2004; Nissen 2010; Nissen 2011; Keshet and Simchai 2014). In this paper I analyse the gender differential in AM consumption and provision against the context of two facts. First, AM use is driven by (a) dissatisfaction with scientific medicine (SM) (McIntosh and Ogunbanjo 2008), (b) the favouring of a more equitable patient–provider dynamic which centres patient autonomy (Gollschewski et al. 2008; Hall et al. 2012), (c) its association with particular ideologies, notably feminism (Astin 1998; Scott 1998). Second, SM does not adequately serve women’s health needs, which are often under-researched (e.g., Mikhail 2005; Holdcroft 2007) and under-treated (Annandale and Hunt 2000; Hoffmann and Tarzian 2001), while women’s bodies are over-medicalized (e.g., Drew 2003; Leidy Sievert 2003). I argue that we should combine these facts and take seriously the idea that women’s attraction to AM is related to the fact that SM neglects women’s needs. This has moral consequences, since AM therapies have a weak or absent evidence base and do not have plausible, verifiable mechanisms, which leaves AM patients under-served, vulnerable to exploitation, and unable to realize meaningful patient autonomy.

This article is structured as follows. In section 2, I explore the claim that SM neglects the needs of women. In section 3, I define AM and describe concerns about its use. In section 4, I describe the typical profile of an AM user and review some common reasons for preferring alternative therapies. In section 5, I argue that despite being chosen in reaction to the shortcomings of SM, AM cannot offer greater patient autonomy and is perhaps more liable to be exploitative. In section 6, I conclude that SM should be reformed to better meet the needs of women and other marginalized groups and recommend that medical professionals prioritize longer consultations for those with long-term and/or unresolved health issues.

## How Medicine Underserves Women

Scientific medicine has a chequered history, particularly in relation to its treatment of women and people of colour in the contexts of theory, research participation, and clinical consultation. Like other forms of science, medicine is value-laden. Its social power renders it prone to reflect the mores of any given era, including the moral failings. Accordingly, certain medical advances may have occurred *because of* the ethical shortcomings of medical practice and research, rather than in spite of them. For example, experimentation on enslaved women in the nineteenth century (Ojanuga 1993), including surgery without anaesthetic, led to important discoveries which ground modern gynaecology. Had this experimentation been deferred until consenting subjects were available—presumably when safe, reliable general anaesthetics were accessible many decades later (Robinson and Toledo 2012)—the progress of gynaecology would have been delayed. This and other abuses of people of colour in medical experimentation have led to reduced trust in physicians (Randall 1995; Boulware et al. 2016) and medical research (Rajakumar et al. 2009) within communities of colour. That the exploitation of certain groups has been critical to medical progress raises questions about who has been centred, and who side-lined, in the development of modern SM.

Women’s confidence in SM continues to be tested, as medicine is often inadequate in meeting the differential needs of female bodies and women patients. Women are still often perceived as “reproductive beings”(Langer and Fleck 2013), which leads to non-reproductive health problems being under-researched and under-diagnosed (see, e.g., Mikhail 2005), while reproduction is over-medicalized (Cahill 2001). For example, while there have been significant advances in artificial reproductive technologies, non-reproductive health issues that are important to women’s well-being or pleasure, such as menopause (Posner 1979; Cordingley et al. 2009) and female orgasm (Tuana 2004), remain under-researched.

Female participants have long been excluded as research subjects (Holdcroft 2007; Liu and Mager 2016), leading to lacunae in specificities of disease in females. Health conditions which affect both sexes can manifest in particular ways in female patients (e.g., anaemia, osteoporosis, cardiovascular disease), rendering standard treatments suboptimal or unsafe (Gronowski and Schindler 2014). Likewise, pharmaceuticals whose safety and efficacy are inferred from studies on male patients are sometimes unsafe and/or ineffective for female patients (Bies et al. 2003; Regitz-Zagrosek 2012).

Gendered inequalities in healthcare are also widely reported in clinical encounters, as physicians make assumptions about women’s health based on gender stereotypes about pain thresholds and patient credibility, while drawing on male-centred research and guidelines (van Wijk et al. 1996; Annandale and Hunt 2000). Women’s pain reports are often discredited or their pain inappropriately attributed to mental health issues (Hoffmann and Tarzian 2001). Women admitted to hospitals in the United States are 13 to 25 per cent less likely than men to receive opioid analgesia for abdominal pain even given the same pain score, wait longer than men to receive any pain medication at all (Chen et al. 2008), and are less likely to receive appropriate treatment for myocardial infarction, leading to a higher mortality rate (Pelletier et al. 2014). These disparities have also been established in relation to race: Black patients are less likely to receive treatments for myocardial infarction than white patients, with Black women faring worst of all, with the highest resultant mortality rate (Vaccarino et al. 2005). Likewise, women with septic shock are more likely to die in hospital than men (Pietropaoli et al. 2010), and critically ill women over the age of fifty are less likely than men to be admitted to intensive care units and receive life-saving treatments, again leading to a higher mortality rate (Bierman 2007).

In general, women’s health issues are liable to be attributed to psychological or emotional, rather than physical, causes. A study investigating gendered differences in the treatment of irritable bowel syndrome found that female patients were more likely to be offered sedatives and lifestyle advice, while male patients were more frequently offered X-ray imaging of the colon (Hamberg et al. 2004). Women are also more likely to have negative experiences with healthcare providers, including not being believed, not being listened to, and feeling they are treated disrespectfully (Upmark et al. 2007).

It is telling that medically unexplained disorders are more common in women (Nimnuan et al. 2001). Their resolution may be thwarted by the disempowerment women patients experience within consultations, leading to a suboptimal assessment of their symptoms (Malterud 2000). Iatrogenic harms through unnecessary investigations are a common outcome (Page and Wessely 2003). Recent research suggests that doctors’ failure to understand medically unexplained symptoms may relate to medicine’s inability to accommodate physical abnormalities whose origins lie in the social stressors associated with being a woman (Herrman 2017). Others understand medically unexplained disorders to be the contemporary version of “hysteria,” a trivializing catch-all for the underfunded, under-researched health conditions which affect women (Jutel 2014).

Medicine, as a site of social power, reflects the contours of power in society at large, and patriarchal values are prevalent in healthcare provision as a structural trend which is also mediated through injustices within individual encounters (Govender and Penn-Kekana 2008). Women’s health testimony is deemed to be less credible, knowledge about both women’s health issues and the specificities of disease in females are inadequate, and women receive inadequate treatment for a range of health problems, leading to higher morbidity and mortality. In the next section I suggest that it may be partly in light of the sluggish pace of change of patriarchal values within medicine that women are over-represented amongst those seeking therapies outside the biomedical paradigm, in search of healthcare that avoids these negative associations and which places greater emphasis on patient autonomy within an equitable patient–provider relationship.

## Defining Alternative Medicine

There are few satisfactory definitions of AM. It is often confused, conflated, or conjugated with complementary medicine, defined operationally (Wieland et al. 2011) as a disparate conjunction of practices, or distinguished from SM only through contrast to it.

First of all, I will redefine and set aside “complementary medicine” in an attempt to simplify an opaque set of uses. I take the term at face value: “complementary” medicine will here refer to therapies that are not exclusive to the medical domain but are nonetheless often endorsed by medical practitioners, since they are complementary to, which is to say facilitative of, good health. Complementary medicine therefore comprises all therapies or heuristics which complement SM but do not attempt to replace it. These are therapies which may be related to one’s lifestyle or obtained through one’s social relations: a healthy diet, exercise, massage, relaxation techniques, group or individual counselling. In other words, complementary medicine tends to involve engagement with *determinants* of health problems, rather than health problems themselves. These interventions generally have an evidence base, even though their effects are often incremental and long-term. For example, massage therapy has been shown to reduce pain, anxiety, and blood pressure (Moyer et al. 2004), while the breathing techniques used in yoga have also been shown to reduce blood pressure (Grossman et al. 2001). Complementary therapies do not generally require medical supervision or facilitation, yet their benefits may be straightforwardly rationalized in line with SM, albeit generally in indirect terms.

AM, on the other hand, departs dramatically from the paradigms of science. AM is used as a replacement for SM, in part or in whole; that is, it sets out to treat ailments that would generally be considered to be medical in nature. Drawing on recent work by Shahvisi (2016), AM^1^ may be defined as intended treatment that:

1. Does not have any proven effect beyond placebo, and;
2. Either:

- does not posit any mechanism;
- posits a physical mechanism that is implausible with respect to known science;
- posits a mechanism that is not physical.

SM therapies elude this categorization on two fronts: as a general rule, they have been proven to be effective beyond placebo and their mechanisms are known and are consistent with the remainder of our science. There are some exceptions and caveats to this claim, which must be spelled out in order to defend against a possible objection. For some SM therapies, such as aspirin and cancer chemotherapy (Shahvisi 2016) there is no firm consensus as to the mechanism. Yet it is important to note that this is a matter of over-determination; the lack of consensus derives from the fact that these therapies have multiple likely mechanisms, all of which are consistent with accepted science. It is just not yet clear which of these is the most determinative of the observed therapeutic effect. There is every reason to believe that the matter will be settled in due course, and no expectation that the ultimate mechanism will be anti-scientific. Regardless, almost all of these therapies have been shown to be effective beyond placebo. This is not to deny that the evidence for common SM therapies can be a complicated matter: some therapies enter general usage without a robust evidence base (albeit exceeding placebo) (e.g. Davis et al. 2017), while therapies emanating from highly cited studies sometimes continue to be used and promoted even after they have been contradicted (Tatsioni, Bonitsis, and Ioannidis 2007). Whilst SM is regulated and particular evidential standards are supposed to be met, the reality is less than perfect (Epstein 2017). Further, while SM therapies are effective beyond placebo, the heavy reliance on placebo within medicine must be acknowledged. Not only do many doctors regularly knowingly prescribe placebos in order to appease patients whose problems are not serious or not easily treatable (Tilburt et al. 2008; Howick et al. 2013), but placebo is an important part of perceived symptom relief for SM therapies (Kaptchuk and Miller 2015).

Homeopathy is perhaps the most famed variant of AM and serves as an expository archetype. Its ostensible mechanism of action is elaborate and internally consistent. It imbues water with a “memory” which outlasts multiple dilutions of an infinitesimal amount of active ingredient. However, not only does this mechanism fail to cohere with the remainder of our vast scientific knowledge but its claims are directly contradicted by that body of knowledge. All of our accepted science tells us that water does *not* have a memory, which means that the tiny amounts of active ingredient in the original solution could not leave a trace in the eventual product, from which the active ingredient is itself absent. Homeopathic pills are therefore just sugar pills, and their efficacy is capped at the nominal placebo effect which applies to all treatments (Ernst 2002; Nuhn et al. 2010).

Consider also chiropractic, which claims that a range of health conditions can be treated through manipulation of the spine. Whilst the details of the proposed underlying mechanisms vary, the central claim of chiropractic is that misalignments or displacement of the vertebrae, known as “subluxations” (which are not necessarily detectable using medical imaging, e.g., X-rays (WHO 2005)), affect the nervous system in such a way as to bring about a range of health conditions, including musculoskeletal pain, headache, asthma, and gastrointestinal problems (Posadzki and Ernst 2011). Chiropractors manually manipulate the spine in ways that are alleged to rectify the subluxations and thereby remedy the associated health conditions. There is no evidence for the existence of chiropractic subluxations (Homola 2010), no scientifically acceptable mechanism for the way in which subluxations cause ill health (Mirtz et al. 2009), and no scientific evidence for the efficacy of chiropractic for the treatment of any medical issue (Posadzki and Ernst 2011).

Likewise, other AM modalities are ineffective beyond placebo and lack mechanisms that are consistent with the remainder of our science, for example, energy medicine (Stenger 1999; Ernst 2006), naturopathic medicine (Atwood 2003), and faith healing (American Cancer Society 2013). Importantly, what all these therapies have in common is the absence of a *plausible* mechanism, that is, one that fits in with the remainder of our collective knowledge and can therefore be verified. As such, their method of action or the reason for their selection cannot be explained to patients, because there is no objective sense in which these facts are knowable by the practitioner, let alone the patient (Shahvisi 2016). The consequences of this difficulty will be returned to in section 5.

## Alternative Medicine as Liberatory?

Drawing on studies across populations in Global North contexts (Australia, Austria, Canada, Denmark, Germany, Italy, the Netherlands, Switzerland, the United Kingdom, and the United States), the profile of a typical AM user is a woman (MacLennan et al. 1996; Verheij et al. 1999; Ernst and White 2000; Stein et al. 2009; Frass et al. 2012) who is more highly educated (MacLennan et al. 1996; Blais et al. 1997; Verheij et al. 1999; Ernst and White 2000; Stein et al. 2009; Frass et al. 2012), relatively affluent^2^ (Blais et al. 1997; Ernst and White 2000; Stein et al. 2009), and often suffering from a long-term health condition (Blais et al. 1997; Busato et al. 2005; Stein et al. 2009; Ernst 2010; Frass et al. 2012).

Empirical research reveals a range of motivations for AM use. Unsurprisingly, users believe that AM is effective in alleviating medical problems (Ernst and White 2000). Amongst other positive motivations, or “pull factors,” are the presumed “naturalness” of AM treatments—the belief that they are “organic,” “pure,” and “safe” (Ernst 2010)—the control they are believed to offer over illness/health (Stein et al. 2009), and the contention that they are person-centred and holistic (Ernst 2010). Negative factors, or “push factors,” also abound: dissatisfaction with SM and the doctor–patient relationship or the clinical encounter (Moore et al. 1985; Mitzdorf et al. 1999; Stein et al. 2009; Ernst 2010) and doubts about the efficacy of treatments within SM (MacLennan et al. 1996; Stein et al. 2009). While many scholars (Furnham and Smith 1988; Joy 2004; McIntosh and Ogunbanjo 2008) conclude that disenchantment with SM is a more determinative indicator for AM use than belief in the efficacy of AM, others (e.g., Astin 1998) determine that a commitment to feminism, environmentalism, or another value-system is most pertinent to AM use. Indeed, various theories have been proposed and tested as to the ideological reasons why people choose AM (Bakx 1991; Astin 1998; Siahpush 1998), which include a desire for greater autonomy, a desire to reduce the power differential between patient and practitioner, and an attempt to access therapies which better fit the patient’s personal philosophy or value system.

Importantly, AM use is associated with greater numbers of encounters with general practitioners and negative associations with SM as a result of iatrogenic effects of long-term medication (Murray and Shepherd 1993; Al-Windi 2004). Further, the relatively high level of education and income amongst service-users indicates that AM is likely to be a considered decision amid multiple options, rather than a last resort. This suggests that patients are preferentially seeking AM where SM *is* accessible, so that even though some of the listed motivations appear to be affirmative of the therapeutic value or ideology of AM, these may also be construed as a tacit critique of SM.

In an empirical study, Siahpush (1998) finds that patients “turn away from orthodoxy not because of its failure to deliver the promise of good health, but because of the way they are treated by doctors.” By contrast, AM is often interpreted as offering a “different conception of social reality and social relations” (Cooter 1988). For many practitioners and users, AM use is a way of expressing resistance to the dominant biomedical paradigm, within which doctors are charged with entrenching the patriarchy of the profession by controlling the information that is legitimate within a medical interaction in ways that uphold their authority (Fisher 1993; Thompson 2003).

Importantly, it has been shown that homeopaths in the United States spend twice as long with patients as their doctors do (Jacobs et al. 1998). AM is seen as being more personalized, and to its users it stands in contrast to SM in that it “does not marginalize or deny human experience; rather, it affirms patients’ real-life worlds” (Kaptchuk and Eisenberg 1998, 1062). Some AM users do not reject SM but rather welcome an opportunity to assume control and autonomy as part of a regime of self-care during chronic illness that is not deemed to be possible within the paradigms of SM (Thorne et al. 2002).

AM is thought to overcome the ontological dualism and one-size-fits-all model that typifies SM. Many AM modalities (e.g. homeopathy) claim to generate therapies in response to the particular needs of an individual patient (Scott 1998). This is deemed to be preferable to the universalism of science, largely because it is intended to optimize the autonomy of the patient. Likewise, midwives using AM in the treatment of childbearing women cite the way in which AM therapies permit greater autonomy for themselves and their patients, which may be a particular reaction to the medicalization of childbirth (Hall et al. 2012).

The preponderance of women clients is only half of the story: AM is also dominated by women practitioners (Andrews et al. 2003; Cant and Sharma 2004; Nissen 2010; Nissen 2011; Keshet and Simchai 2014). In 1998, women students made up 90 per cent of homeopathy classes in the United Kingdom, while almost 80 per cent of those on the U.K. Society of Homeopaths Register were women (Morrell 2007). At the time of writing, women constitute 87 per cent of those registered with the Society of Homeopaths,^3^ 81 per cent of those registered with the U.K. Complementary and Natural Healthcare Council, 4 which regulates a range of forms of AM, 5 and 58 per cent of those registered on the General Council and Register of Naturopaths.^6^ It has been noted that the gender disparity is not so striking for mechanical, more “scientific,” modalities of AM such as osteopathy and chiropractic, which generally require full-time training that may deter women with other commitments (Nissen 2011). Indeed, 50 per cent of those registered with the General Osteopathic Council are women (General Osteopathic Council 2018). (Consider that 54 per cent of doctors in the United Kingdom are men [General Medical Council 2018]). Further, there is evidence of a gendered difference in attitude towards AM amongst medical students across different contexts, with women students more likely to view alternative therapies positively (Furnham and McGill 2003; Lie and Boker 2004; Greenfield et al. 2006; Akan et al. 2012).

Women’s dominance as users and service-providers within AM can be contextualized within a broader trend of women’s greater interest in spirituality and the holistic milieu (Houtman and Aupers 2008). In the United Kingdom, women’s production and use of holistic products and services is four times that of men (Heelas and Woodhead 2005). AM care is often assumed to offer attributes that are commonly identified with normative femininity, that is, being caring, being gentle, having strong communication skills, taking emotions seriously, and seeking to care for rather than cure (Shuval and Gross 2008, 51; Keshet and Simchai 2014, 81). The holistic approach typified by many AM modalities is theorized to be attractive to women because it is coherent with, and legitimizes, the relationality that women are socialized to embody in their care-giving but at the same time validates notions of self-care which subvert the stereotypical care role and recognize the importance of a woman “thinking about her own well-being rather than that of her dependents” (Sointu and Woodhead 2008).

There is evidence that feminist values are important within AM, for both users and practitioners. Twelve of thirteen U.K. homeopaths interviewed by Scott (1998) described themselves as feminists, and most of the practitioners had at some point engaged in activism around social justice issues and were in part driven by an active interest in health politics. Scott notes that “feminist health activists have argued that orthodox biomedicine imposes passivity, ignorance, and powerlessness on patients, and particularly on female and non-white patients” (Scott 1998, 195). Three themes are identified as being central to the attraction of “feminist homeopathy”: the power differential in SM, the failure of biomedicine to consider social/contextual concerns, and the trivialization of women’s knowledge and concerns in SM encounters (Scott 1998, 197). Likewise, Shuval and Gross (2008) found that midwives practicing AM in the delivery room justify this choice through aspects of feminist ideology, notably the empowerment of women patients and the rejection of the medicalization of childbirth.

Empowerment and autonomy are repetitive themes in the literature on women’s engagement with AM. In an Australian study of menopausal women, empowerment emerged as the central motivator for choosing AM therapies, with women seeking greater control in relation to their symptoms and the treatment choice, and a desire for better information (Gollschewski et al. 2008). Study participants described themselves as “information seekers” and sought a “better understanding of the changing needs of their bodies, the symptoms they were experiencing, and the therapies available to them” (Gollschewski et al. 2008, 166). They did not feel that their general practitioners were able to provide this and found the patient-doctor relationship asymmetric, with their own experiences often dismissed or trivialized.

Keshet and Simchai discuss the way in which AM treatments are valued amongst users for the way in which they flatten the power imbalance between practitioner and patient, permitting the patient to avoid patriarchal dynamics within SM and instead feel like an authoritative, active partner in the exchange (2014, 82).

In short, AM is valued both for pragmatic medical reasons—because it is believed to be effective, it is assumed to have few or no side effects, it is deemed to be more “natural”—and for ideological reasons—because it is believed to offer control over health, be non-authoritarian, and improve patient autonomy by avoiding paternalism and universalism. It is easy to see why AM may be an attractive possibility for women whose health needs are not met by SM. In the next section, I will show that the presumed virtues of AM are, for the most part, not supportable, and the resulting liberatory credentials are undeserved. This is largely because the therapies that fall within AM have never been proven to be effective beyond placebo. But much more worryingly, I will show that patients cannot give informed consent to therapies whose mechanisms cannot be explained to them in good faith.

## Alternative Medicine as Paternalistic

Perhaps the most famous feminist health tome is *Our Bodies*, *Ourselves*, whose 1978 edition proposed twenty rights which hospital patients ought to be able to claim (Boston Women’s Health Book Collective 1978). Half of them pertain to a patient’s right to be fully informed regarding both the condition that is being treated and the treatment that is being offered. This is reflective of the importance of autonomy to women’s health interactions.

Feminists have been critical of the centrality of autonomy to philosophical conceptions of personhood, since in its standard form it is deemed to be contrary both to the interests of women, whose lives are strongly characterized by relationality, and to the interests of society as a whole, where relationality should be acknowledged and valued (e.g. Jaggar 1983). Autonomy-focused descriptions of personhood tend to idealize atomistic, self-sufficient agents, which is judged to be both descriptively false and normatively problematic with respect to social beings. More recently, autonomy has been recuperated by feminists, as it has been recognized as a critical apparatus for understanding the way in which patriarchal structures tend to over-rule and undermine women’s autonomy (Stoljar 2013). Recuperated versions are often more accurately described as “relational autonomy,” where due regard is paid to relationships with others and the effect of external limiting factors, such as distributions of power (Mackenzie and Stoljar 2000; Narayan 2002; Ells 2003).

Autonomy is particularly important to feminist thought in the domain of healthcare, because demanding autonomy is the most obvious and direct way of avoiding paternalism. Paternalism occurs when the autonomy of a competent person is overridden on the grounds that an action is intended to improve their well-being. The risk of paternalism in healthcare is significant because of the power dynamic between a person in need of treatment and a person who is able to offer treatment within an establishment that is considered to be authoritative (Goodyear-Smith and Buetow 2001).

Within medical settings, healthcare professionals endeavour to avoid paternalism by securing informed consent from all patients before issuing any treatment. In the ideal case, informed consent is obtained by providing the details of the treatment options to the patient in such a way as to ensure that she is able to understand the reason for a particular treatment being recommended and is then invited to approve that treatment choice (Shahvisi 2016). In other words, the patient ought to leave the consultation understanding why she has been given antihistamines rather than antibiotics (say), as the endpoint of a transparent, collaborative reasoning process between doctor and patient. In practice, this ideal is rarely met, since, amongst other limitations, health professionals are subject to time constraints, patients’ scientific knowledge is usually limited, and many patients trust the medical establishment and accordingly shortcut the process by deferring to their doctor’s authority (see e.g. O’Neill 2003). Whilst these limitations are concerning, it is important to note that ideal informed consent is not an impossibility in these contexts.

By contrast, AM therapies cannot deliver on the demand for ideal informed consent, and this limitation is a matter of principle, not practice. It seems that AM is therefore *necessarily* paternalistic. The inescapability of this paternalism operates in two independent ways. First, no scientifically acceptable explanation can be given for the mechanism of the therapy, which means that exemplary informed consent is ruled out. Second, should too much information be given about the therapy, its nominal positive effect—which relies on the placebo effect, which in turn relies on belief that a therapy is efficacious—may be threatened.

In other words, AM practitioners necessarily cannot obtain genuine informed consent from their patients in the way that SM practitioners aspire to. AM therapies cannot be recommended to patients by reference to their mechanisms without asking patients to accept explanations which are not consistent with the vast body of knowledge we collectively accept. Nor can they be recommended on the strength of their evidence base, since none has been shown to be effective beyond placebo.

To approach this argument another way, consider O’Neill’s suggestion that genuine informed consent requires that a patient is “neither coerced nor deceived, and can judge that they are not coerced or deceived,” and that patients are offered “extendable information,” which is to say, “easy access to more specific information that lies behind an initial, or second, or third layer of information provided” (2003, 6). Two points must be made. First, I am not claiming that AM practitioners deceive their patients, deliberately or otherwise, though that may sometimes be the case. Rather, I am suggesting that they do not meet O’Neill’s requirement that they could, in principle, be exposed as deceitful. Consider that we make judgements of whether or not we are being deceived by assessing the likely truth of a given proposition. Assessments of truth are made by reference to a set of auxiliary assumptions regarding what we already believe to be true in the world, and consistency with those auxiliary beliefs is key to assessing the truth value of a new proposition. Yet the claims made within AM are *not* consistent with our most basic auxiliary assumptions about the world. (Note that even a person who rejects, or claims to reject, scientific knowledge, still draws liberally on scientific knowledge in their everyday life; scientific knowledge underwrites all our bodily decisions as physical beings.) It is therefore not possible for patients to verify their likely truth unless they already hold the proposition “this practitioner’s treatments will work” within their set of auxiliary assumptions, in which case limitless deceit is possible. Second, extendable information is not possible for AM, precisely because it is not founded on the scientific principles which guarantee an extendable information base for all other medical queries. If someone enters into a regress of why-questions regarding the choice to prescribe methylphenidate for attention deficit hyperactivity disorder, there will be detailed answers available all the way down to the atomic level. Not so if a patient wishes to understand why a chiropractic procedure has been recommended for neck pain. Chiropractic has not been demonstrated to be definitively effective for the treatment of any medical condition, and its ostensible mechanisms are not consistent with accepted science (Ernst 2008; Posadzki and Ernst 2011).

Chatwin presents transcripts of consultations in order to explore the difficulties AM practitioners face when attempting to explain their reasoning and field patients’ questions and challenges. Unable to rely on the persuasive power of conventional explanations or bodies of evidence, practitioners must pre-emptively buttress their credibility through careful management of the therapeutic encounter. Practitioners are often compelled to tactically reinforce the axioms of their therapy early on in the encounter, which means they can subsequently “utilise previously prepared and familiar sequences of narrative in which they are, by default, cast as the figure of authority” (Chatwin 2008, 250). This description stands in stark contrast with the motivations for AM recorded in section 4, where resisting biomedical authority and seeking autonomy within participatory care were paramount for most patients.

It seems that patients must simply *believe* in the proffered AM therapy, because they trust either the practitioner or the paradigm. Indeed, the efficacy of AM treatments relies on this trust and may be undermined by its removal. Yet trust without informed consent is patently a violation of autonomy and seems to suggest a reliance upon unchallenged authority within the AM paradigm, which violates its celebrated virtues of offering autonomy and control to patients. And whilst AM may offer non-universal, personalized therapies, one wonders at the value of these bespoke therapies if their particularities are not explainable in ways that meet our usual standards of compatibility with other bodies of knowledge. While standardized care within SM has its shortcomings, individualized care in the context of AM is vulnerable to charges of arbitrariness.

Further, it is not controversial to suggest that equality and justice in healthcare, as in other spheres of our social world, require engagement with a common substrate of meaning. It would be very difficult to adjudicate charges of racism (say) in AM delivery if there are no benchmarks against which to measure standard, acceptable care, or to be able to predict what a patient might reasonably expect, and why. Avoiding abusing one’s power requires transparency with others, and that transparency can only be obtained if you are able to justify your choices in such a way that the patient can verify that they were appropriate or elect another person to do so.

Of course, none of this militates against the reality that many AM patients are very satisfied with their care. Indeed, most are more satisfied with their AM practitioner than their general practitioner (Finnigan 1991). Undoubtedly, there is much to admire in the therapeutic relationships of AM practitioners, who generally spend longer with patients, hear a more detailed history, and tend to generate an encounter that is perceived to be compassionate and collaborative. Of course, almost all AM is privatized, which liberates practitioners from the constraints on time and resources that are so common in public healthcare systems.

In short, AM’s lack of engagement with plausible mechanisms of action, and ipso facto, with explanations and informed consent, leads to a situation in which there is a therapeutic free-for-all in which all manner of (potentially contradictory) claims may be made, and in which patient autonomy drops off the agenda, leaving those who are marginalized by SM with neither effective treatment nor meaningful autonomy. Where independent verification has no mileage, and trust is so critical, the potential for abuse is considerable. As AM becomes progressively more commercialized (Collyer 2017), this is a grave concern.

SM has done a good deal of damage, but it has also brought about some of our most unequivocal goods. Many of its benefits are unfairly distributed, it is sometimes insensitively delivered, and its operation is too often oblivious to social and political factors, but once these realities are fully apprehended, they become questions of practice, not principle, and do not bear on the potential of the paradigm to bring about positive change. The trouble with AM seems to run much deeper, and it is therefore a matter of moral urgency for those working within SM to ensure that patients’ decision to choose AM therapies is not primarily motivated by disillusionment with medicine, especially as a result of a particular marginalized identity.

## Conclusion

Within this paper I have made the following argument: SM is patriarchal and under-serves women; women dominate AM, both as users and service-providers; women who choose AM often cite dissatisfaction with SM and the desire for greater autonomy and personalization within the clinical encounter. Based on these premises, it is likely that women use AM because it promises to offer greater autonomy and personalization than SM. This segues into a second argument: autonomy in healthcare requires informed consent; informed consent is not possible for AM therapies since mechanisms are either not known or not plausible, and there is no evidence base. These premises entail that AM cannot help patients to realize autonomy. Combining these two conclusions, it seems that AM does not offer the control and autonomy that is sought. Whilst it may be argued that AM offers personalization and a satisfactory therapeutic encounter, it must also be noted that forfeiting an evidence base, plausible mechanisms, and the ability to make autonomous decisions is a heavy loss to patients and one for which SM must take some responsibility.

I have attempted to motivate each of the above premises in the preceding sections. In this section I reflect on the implications of these conclusions and make recommendations to ensure that women’s health needs are more adequately met.

First, to the extent that rejection of SM may be seen as a form of resistance, the cost of that resistance is borne largely by the resistors themselves. Those who begin with a sense of dissatisfaction with SM—in many cases stemming from SM’s failure to provide them with adequate healthcare or to inspire trust in its own decency—end up with healthcare that is on many counts less adequate.

With the ascendance of a post-factual culture, arguments relying on evidence, reproducibility, and consistency are liable to have ever less traction. By corollary, the features that have typically worked against AM—its lack of an evidence base—are likely to pose less of a barrier to its uptake. This ought to be a grave public health concern, since the well-being of entire populations depends on medicine earning and retaining the trust of all. To see this, one need only consider how easily herd immunity is lost when trust in vaccination is undermined, putting entire populations at risk of disease outbreaks (Casiday et al. 2006). Recommending against vaccination is common amongst AM practitioners (especially within chiropractic, homoeopathy, and naturopathy) whose philosophies so often rely on emphasizing, and in many cases overstating, iatrogenic risk (Ernst 2001).

Not much can be done now to atone for medicine’s history except to openly accept its shortcomings and undertake particular efforts to re-engage marginal health populations. While it is easy to suggest that researchers should be devoting more time to women’s health issues and their treatments, the bench-to-bedside timeline is long, and more immediate efforts are also necessary. As Ernst (2010) has noted, concentrating on the therapeutic relationship within SM seems like the most constructive way forward. Although patients often look to SM for the “science of medicine,” clearly many are turning to AM for the “art of medicine” (Ernst 2010, 1473)—for a compassionate clinical encounter in which patients are humanized and power differentials are flattened. While SM may have the upper hand in terms of mechanisms, an evidence base, and social capital, there are inadequacies in the patient–practitioner relationship, and in this respect there is much to learn from AM modalities, where the therapeutic relationship is key to their appeal. Even the most resolute AM user inevitably encounters SM professionals on occasion. Provided broader pressures on health workers permit them the time and space, those encounters present the possibility of demonstrating that SM can be person-centred, equitable, and sensitive to the differential needs of marginalized patient groups.

As I described in section 4, many of those who choose AM do so following a long period of unsatisfactory encounters with medical professionals as they pursue the treatment or resolution of long-term chronic ailments (Furnham and Vincent 2000; Cant and Sharma 2004). The majority of these patients are women. While AM practitioners are unlikely to offer therapies that are effective beyond placebo, they are able to offer consultations which are longer, more participatory, and more personalized. It is likely that most of the placebo effect is interpersonal and stems from the ritual of healing within encounters with practitioners, rather than from any specific therapy (Miller et al. 2009). There is some evidence that open label placebos still confer a placebo effect, which suggests that the therapeutic relationship plays a significant role (Kaptchuk et al. 2010). In light of these insights, Blease (2012) suggests that the placebo effect instead be referred to as the “positive care effect.” Given the importance of communication and autonomy amongst patients choosing AM, it is interesting to note that a surgical study shows that the extent of the positive care effect is contingent on the quality of the clinical encounter, with communication skills aimed at empowering patients being predictive of better clinical outcomes (Trummer et al. 2006).

Focusing on the U.K. healthcare system, I therefore recommend that general practitioners, who are the gatekeepers of the medical profession, make efforts to address the inadequacies in the clinical encounter, specifically for those with long-term health conditions or medically unexplained symptoms. As it stands, general practitioners in the United Kingdom spend ten minutes with each patient and are encouraged to focus on a single health issue. It is therefore unsurprising to note that dissatisfaction with the clinical encounter is shared by clinicians. In a recent survey, 55 per cent of general practice surgeries in the United Kingdom reported concerns about the quality of care they could provide and described their workload as unmanageable most of the time; 13 per cent reported that it was unmanageable all of the time (Iacobucci 2016). In another study, 68 per cent of general practitioners expressed the view that care could be improved by longer, higher-quality consultations, while 67 per cent felt that patients with long-term conditions should be afforded longer consultations (Rimmer 2015). It has been demonstrated that longer consultation times correlate with a greater likelihood of taking a thorough medical history and conducting examinations in accordance with good practice, a lower prescribing rate, a greater likelihood of offering advice about preventative healthcare, and fewer follow-up consultations (Wilson and Childs 2002).

General practitioners are currently able to make referrals to specialists in various clinical disciplines. In addition to lengthening standard consultation times, the option of making general referrals may be a constructive way forward, i.e. arranging for the patient to have a lengthier consultation with a general practitioner rather than being siloed into a specialist referral (which is liable to be an even less holistic encounter) or sent away. Given the importance of the therapeutic encounter, it is also worth considering increasing the number of talking therapies referrals for long-term physical health problems. As it stands, talking therapies are recommended within the U.K. National Health Service for a range of social, mental, and physical conditions. This could be broadened, so that those whose physical symptoms are not being satisfactorily resolved within the biomedical paradigm are able to benefit from a personalized therapeutic relationship which does not rely on implausible mechanisms (NHS Choices 2016).

That women may be less likely to benefit from medicine and therefore more likely to spend time and money seeking therapies whose claims are questionable, whose benefits are negligible, and whose potential for exploitation is considerable, is a grave matter. Researchers and clinicians must take responsibility by consciously modernizing biomedicine to ensure that its goods are accessible to all and that the benefits of a positive therapeutic encounter are acknowledged and prioritized in the delivery of care.

## Footnotes

1 Hereafter, I will only refer to alternative medicine, as defined here, as this is the target of my analysis. This presents an apparent methodological difficulty, since the literature upon which I draw does not use a consistent definition of alternative medicine. Very often, studies coalesce complementary and alternative medicine into the same term (as “CAM”), or, where definitions of either are attempted, there is variability, and none quite matches my own. In practice, this doesn’t present a serious difficulty, since it is standard for each paper to also offer an operational definition (by listing treatments of interest), from which one can confirm that the therapies in question are (largely or solely) those which in any case qualify as alternative medicine according to my definition, even if many of them use the looser and more customary “complementary and alternative medicine” label.

2 It is important to note that while wealthier people are the primary users of AM in Western contexts, elsewhere this trend is reversed. In India and Nepal, poorer, less empowered groups tend to be the primary clients of AM practitioners, since AM is a cheaper, less technologized sector, which is therefore more accessible to those with fewer resources (e.g., women, older people) (Broom et al. 2009; Cameron 2010).

3 Pamela Stevens, Marketing Manager of the Society of Homeopaths (pers. comm. May 17, 2018).

4 Margaret Coats, Chief Executive and Registrar of the Complementary and Natural Healthcare Council (pers. comm. May 17, 2018).

5 Despite its name, the therapies regulated by the Complementary and Natural Healthcare Council are almost entirely those defined as alternative medicine in section 3 of this article. I list each of them here, along with a reference which demonstrates the paucity or absence of scientific evidence for the efficacy of the therapy: Alexander technique (Baggoley 2015; NHS Choices 2015), aromatherapy (Baggoley 2015), Bowen therapy (Baggoley 2015), colon hydrotherapy (American Cancer Society 2015), craniosacral therapy (Atwood 2004; Hartman 2006), hypnotherapy (Flammer and Bongartz 2003), acupuncture (Ernst 2009a; Colquhoun and Novella 2013), naturopathy (Atwood 2003), reflexology (Ernst 2009b), reiki (Lee et al. 2008), shiatsu (Robinson et al. 2011). In addition to these, the following evidence-based complementary therapies are also regulated by the same body: nutritional therapy, sports therapy, massage therapy, and yoga therapy.

6 Lisa Smith, General Council and Register of Naturopaths Registrar and Secretary (pers. comm. May 18, 2018).
